# Generation of perfect vortex and vector beams based on Pancharatnam-Berry phase elements

**DOI:** 10.1038/srep44096

**Published:** 2017-03-09

**Authors:** Yachao Liu, Yougang Ke, Junxiao Zhou, Yuanyuan Liu, Hailu Luo, Shuangchun Wen, Dianyuan Fan

**Affiliations:** 1 Laboratory for Spin Photonics, School of Physics and Electronics, Hunan University, Changsha 410082, China

## Abstract

Perfect vortex beams are the orbital angular momentum (OAM)-carrying beams with fixed annular intensities, which provide a better source of OAM than traditional Laguerre-Gaussian beams. However, ordinary schemes to obtain the perfect vortex beams are usually bulky and unstable. We demonstrate here a novel generation scheme by designing planar Pancharatnam-Berry (PB) phase elements to replace all the elements required. Different from the conventional approaches based on reflective or refractive elements, PB phase elements can dramatically reduce the occupying volume of system. Moreover, the PB phase element scheme is easily developed to produce the perfect vector beams. Therefore, our scheme may provide prominent vortex and vector sources for integrated optical communication and micromanipulation systems.

Orbital angular momentum (OAM) has been identified as a useful degree of freedom for enhancing the information carrying capacity of photon because of its unlimited dimensions^1^, and also has been found to have broad applications in optical manipulation and metrology due to the singularities in phase and intensity^2^. Laguerre-Gaussian (LG) beam is the most widely studied optical carrier of OAM as its helical phase (*exp(ilφ*), *l* is the topological charge, and *φ* is the azimuthal angle) contributes to a finite OAM per photon (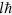, 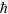 is the reduced Plank’s constant 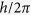)^1^. However, general LG modes are characterized with an annular intensity varying with the change of topological charge, which inevitably confines the co-propagation of multiple OAM modes in communication systems. Thus, a reliable source providing OAM-carrying beams with fixed size of intensity would be meaningful. Moreover, a OAM source with large topological charge and small size of ring-pattern intensity is generally expected for trapping and manipulating the nano-particles.

Perfect vortex (PV) beams provide a solution to this problem^3^, which keep the size of intensity pattern no matter what the topological charge is. Theoretically, PV beam is the Fourier transformation of a Bessel Gaussian (BG) beam^4^. Plenty of schemes have been proposed to obtain the PV beams including using spatial light modulator^3^,^4^,^5^,^6^,^7^, axicon^8^,^9^, interferometer^10^, and micro-mirror devices^11^. Optical manipulation based on the tightly focused field of PV beams has also been extensively studied^8^,^12^. The utility of particle trapping and high efficient OAM transfer promise a broad application scope of PV beams. Furthermore, the non-diffraction property of PV beams scattering field shows the potential use in imaging and cryptography^13^. However, most of these methods are dependent on cumbrous reflective devices or bulky refractive devices to produce PV beams, which undoubtedly limits the application of PV beams in miniaturized optical systems and in fiber communications.

In this work, we designed planar Pancharatnam-Berry (PB) phase elements to generate PV beams. The evolution of polarizations in optical field will introduce an additional phase item in wave function, which is referred as the PB phase^14^,^15^. PB phase optical elements are dramatically developed in recent years due to their exceptional property of phase engineering, which provide a convenient approach to construct highly-functional planar devices^16^,^17^. Arbitrary phase distributions can be pursued and the phase responses of different incident circular polarizations are constantly contrary in a PB phase element. Therefore, PB phase elements including optical lens^18^, vortex beam generators^19^,^20^,^21^, hologram devices^22^, mode transformer^23^, and OAM multiplexer^24^ have been realized up to now. By designing and assembling novel planar PB phase elements, a simplest scheme to produce PV beams is presented in this work. Moreover, our scheme is developed to generate the perfect vector beams, which possesses also the fixed annular intensity but is endowed with azimuthally varying polarizations.

## Results

### Theoretical analysis

Perfect vortex beams are firstly considered as an ideal model of vortex beams with constant intensity distribution which doesn’t depend on its topological charge^3^. Dirac delta function was applied to describe these beams as the following form:


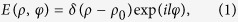


where (*ρ, φ*) are the polar coordinates in beam cross section, *l* is the topological charge, and *ρ*_0_ is the radius of annular bright intensity. However, in general cases, this model is not accessible in experiment. Thus, an annual pattern with small width Δ*ρ* is developed to approximate the above model, which can be described as


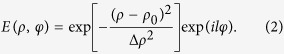


Mathematically, the approximate model of PV beams can be deduced from the Fourier transformation of an ideal Bessel beam function^4^, which can be expressed as


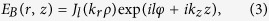


where *J*_*l*_ is the first kind of *l*-th order Bessel function, 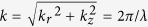, *r* = (*ρ, φ*), and (*k*_*r*_, *k*_*z*_) are the radial and longitudinal wave vectors respectively. However, this mathematical model is also an idealization of PV beams. For most part of practical situations, the accessible Bessel model is actually a BG beam:


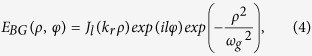


where *ω*_*g*_ is the beam waist to restrict the total field. Many approaches have been exploited to generate such BG beams. Axicon is the most widely applied refractive element^25^, which could convert a general Gaussian beam or LG beams to the zeroth or higher-order BG modes.

According to the Fourier transformation theory, the transformed field of a BG beam can be derived as


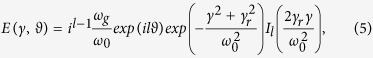


where *ω*_0_ = 2*f*/*kω*_*g*_ is the Gaussian beam waist at focal plane, *f* is the focal length, *I*_*l*_ is the *l*-th order modified Bessel function of the first kind. It is clear that a modified Bessel function and a Gaussian function are conjoined to shape the amplitude, which contribute to the maximum value at *γ* = *γ*_*r*_ and the sharp reduction of amplitude beyond the range *ω*_0_. Thus, a vortex beam with fixed annular intensity (radius equals *γ*_*r*_ and width equals 2*ω*_0_) is constructed, which is namely the PV beam. The value of annular radius *γ*_*r*_ depends on the radial wave vector, which can be modulated by the parameter of axicon (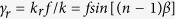, where *n* is the refractive index and *β* is the base angle of axicon).

**Figure 1:**
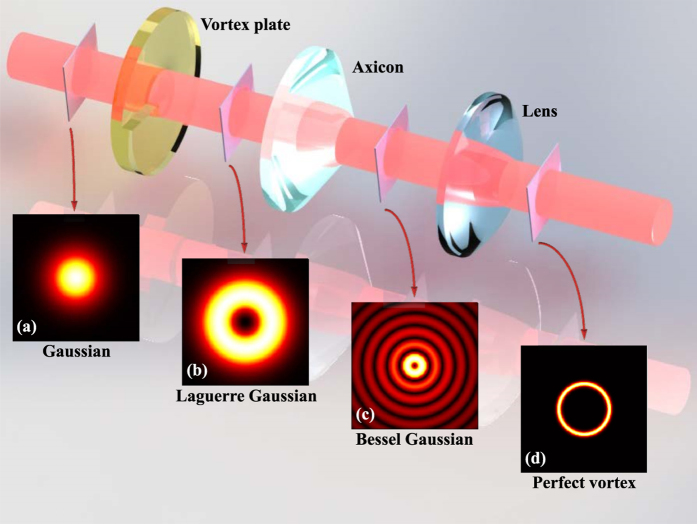
The conventional optical system applied to generate PV beams, which is based on the bulky refractive devices. Combination of vortex plate and axicon convert a general Gaussian beam to BG mode. Then, the lens implements Fourier transformation of BG beam to get the PV beams. Insets show the intensity patterns in each step of this system.

Figure 1 demonstrates a set of refractive apparatus for producing PV beams. A vortex plate constructed with increasing thickness along azimuthal direction, which gives beam the phase variation from 0 to multiple 2*π*, will transform a general Gaussian beam (Inset (a) in Fig. 1) to higher-order LG beam^26^, as the example shown in inset (b) of Fig. 1. Then, an axicon possessing conical surface is applied to obtain the corresponding BG mode (Inset (c) in Fig. 1). Finally, a plano-convex lens is used to implement the Fourier transformation and the PV beam is produced as shown in inset (d) of Fig. 1.

**Figure 2:**
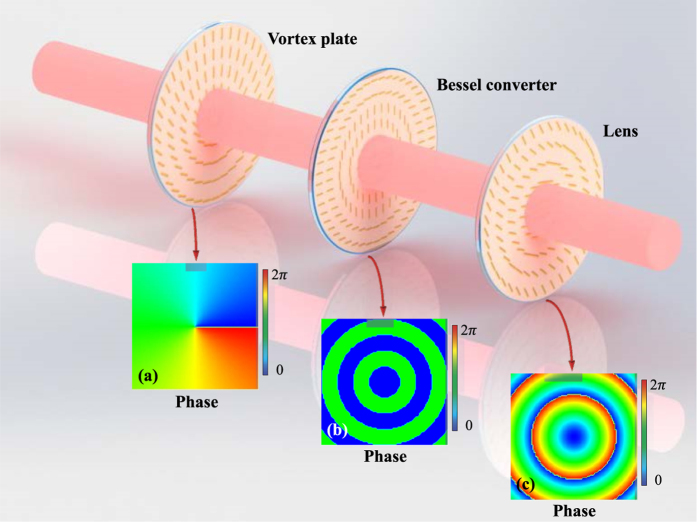
The PB phase element system used to produce PV beams. Planar vortex plate and Bessel converter are combined to generate a higher-order BG mode. Following lens transfers this mode to correspond PV beam. Comparing with the system exhibited in Fig. 1, PB phase elements will dramatically reduce the occupying volume of the entire system. Insets show the phase distributions of each element respectively.

To replace all the elements involved in the conventional refractive system, we should firstly find out the phase profiles imposed by these elements. Moreover, comparing with using axicon in producing the BG beam, there is a simpler scheme by designing a planar device to introduce the derived phase from Bessel function (as shown in inset (b) of Fig. 2)^4^. Thus, the phase distributions corresponding to vortex plate Φ_*V*_, Bessel converter Φ_*B*_, and lens Φ_*L*_ can be defined as


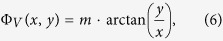



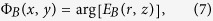



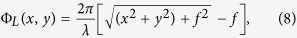


where (*x, y*) is the Cartesian coordinates in beam cross section, *m* is the topological charge of desired mode, *E*_*B*_(*r, z*) is the function of Bessel beam presented in Eq. 3 when *l* = 0, and *f* is the focal length of lens. Examples of phase distributions (m = 1) are demonstrated in insets of Fig. 2.

Pancharatnam-Berry phase elements are widely studied in recent years as their responses can be locally adjusted by rotating the orientation of building blocks. When a PB phase element is constructed based on the local anisotropic response of blocks, the geometric phase obtained in a circular polarization incidence can be concluded as Φ = ±2*ϕ*^18^, where the sign depends on the helicity of the circular polarization, and *ϕ* refers to orientation local optical axis. Thus, arbitrary phase devices can be contrived according to this relationship. The axis orientations of vortex plate, Bessel converter, and lens are deduced as


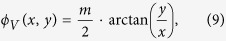



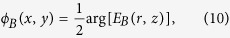



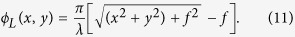


Figure 2 shows the schematics of these PB phase elements and their assembly to produce PV beams. Short lines in each plate suggest the orientation of local optical axes. Comparing with the conventional case, the PB phase system is simplified as the great reduction of thickness of each element.

### Experimental results

**Figure 3:**
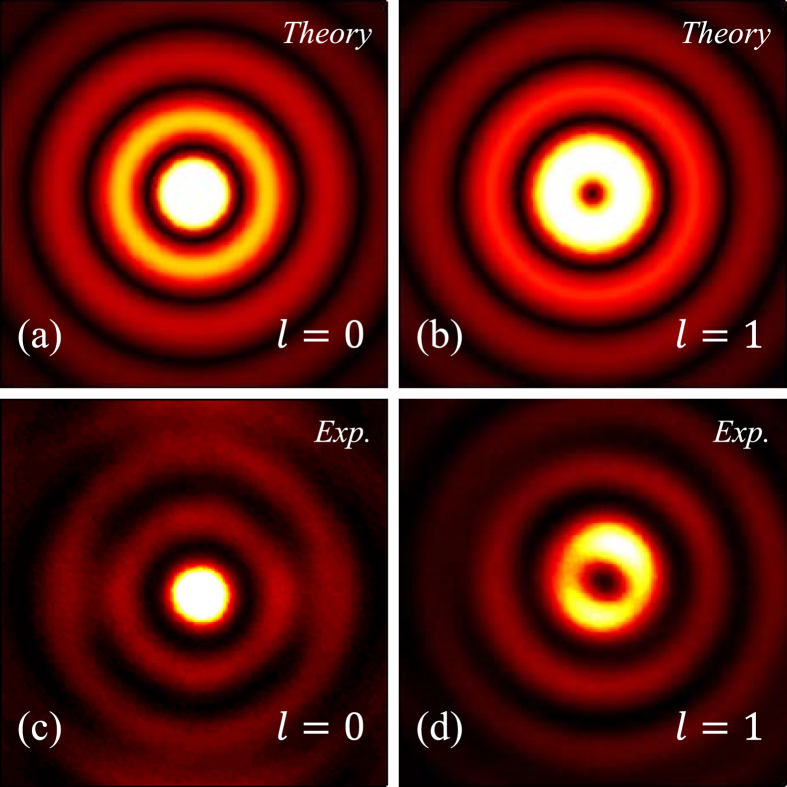
Intensities of zeroth (left column) and first order (right column) BG modes obtained by using planar PB phase elements. First line and second line show the theoretical and experimental distributions respectively.

By using the PB phase elements designed above, BG beams and PV beams were produced in our experiments. The combination of PB phase vortex plate and Bessel converter produced high quality BG beams in experiments as presented in Fig. 3. Theoretical intensities are obtain according to Eq. (4) where *ω*_g_ and *k*_*r*_ are well selected (as shown in the upper row of Fig. 3). Experiment results of two different orders *l* = 0 *and* 1 are demonstrated in the lower row of Fig. 3): the first one was produced by the single PB phase element constructed according to Eq. (10)23-Feb-2017 16:22, of which the optical axis distribution are exhibited as the second plate in Fig. 2; the second one was produced by the combination of vortex plate (according to Eq. (9)) and the zeroth order BG plate. Incident light was set to be circular polarization (either right- or left-circular polarizations) by a combination of polarizer and quarter-waveplate.

**Figure 4:**
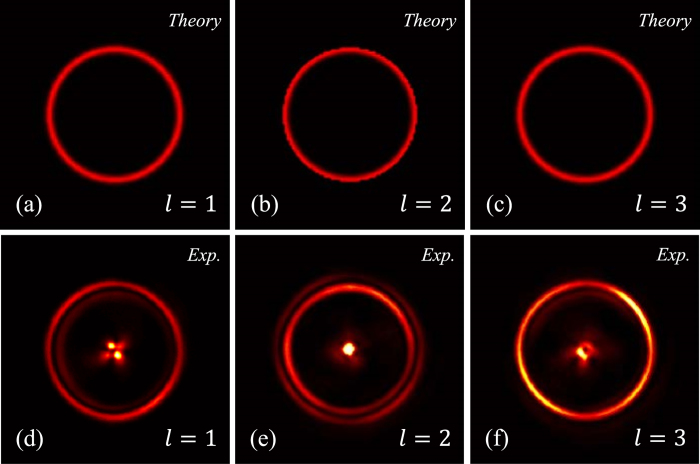
The PV beams generated in experiment. Three different topological charges, *l* = 1, 2, and 3 are chosen. First line shows the theoretical patterns, and second line shows the experimental results respectively. The same as theoretical predictions, radiuses of observed annular intensities are independent on the topological charges.

Then, the Fourier transformation was implemented by the PB phase lens (according to Eq. (11), where *f* are chosen as 200 *mm*) to get PV beams. Annular intensities were observed on the focal plane as shown in Fig. 4. Different results of topological charges *l* = 1, 2, and 3 are demonstrated. The upper row shows the intensities calculated according to Eq. (5) and the lower row is the corresponding experimental results. The radiuses of recorded annular intensities are unvaried when changing the topological charges. Defects located at the center of patterns are inevitable because a portion of diffused light was focused by the exit lens.

## Discussion

The advantage on the PB phase system over traditional system in obtaining PV beams is not only the reduction of thickness of the whole system but also the promising prospect in integration applications. All these three elements can be once fabricated in a single glass thus to provide a PV source in integration photonics and plasmonics. Moreover, as the contrary phase responses of orthogonal circular polarization states can be introduced at the same time, our PB phase system can be directly adapted to produce the perfect vector beams.

Cylindrical vector beam (CVB) is the axially symmetrical solution of vectorial electromagnetic field Maxwell’s equations^27^, which have shown great potentials in particle trapping^28^, plasmon excitation^29^, microscopy^30^, high resolution imaging^31^,^32^, high capacity information coding^33^, and laser processing^34^. Generally, the intensity of CVB is endowed with a LG profile. However, vectorial fields corresponding to other intensity profiles may provide the additional exotic properties. For examples, vector BG and Hermite-Gaussian have been studied recently to show their distinctiveness^35^,^36^,^37^. PV beams provide excellent resistance to the variation of topological charge and the perturbations in propagation, thus, the CVB with PV profile will be fervently desired in applications.

**Figure 5:**
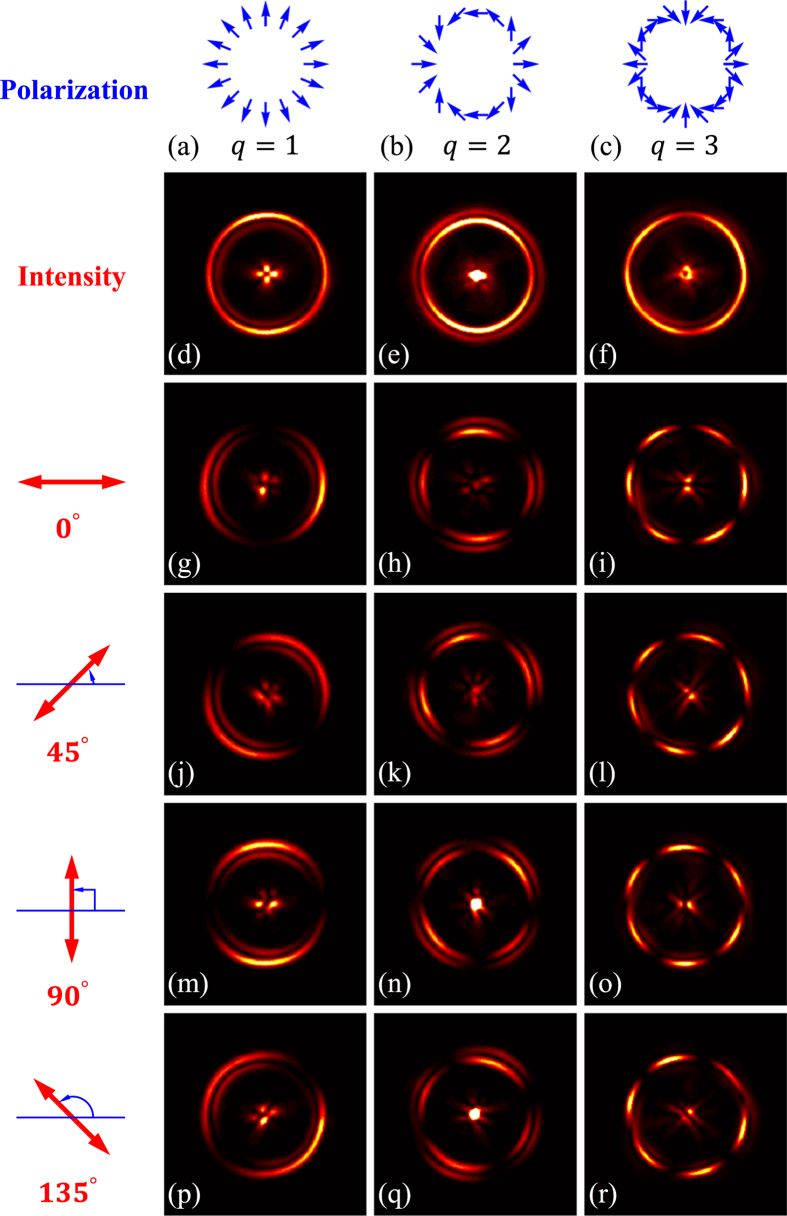
The perfect vector beams generated in our experiment. Three different polarization orders *q* = 1, 2, and 3 are displayed from left to right. First line shows the schematic polarization distributions (blue arrow diagrams), of which the experimental results are presented in each column respectively. Second row is the observed intensities which show the similar annular patterns as corresponding PV beams. Following rows are the polarizer examined intensities to verify the polarization distributions. Different axis orientations of polarizer 0°, 45°, 90°, and 135° (red arrows in the first column) are selected in experiment. Well agreements suggest that the obtained perfect beams are CVB, namely the PV beams here.

Cylindrical vector beam has been proved to be the superposition of two orthogonal circularly polarized vortex beams with opposite chirality^38^,^39^. By changing the incidence to linear polarization, which is the equal superposition of orthogonal circular polarizations, the combination of PB phase vortex plate and Bessel converter will produce a vector BG field. However, the PB phase lens will terminate this adaption because contrary radial phase gradients will be obtained by the opposite incidences. Thus, a general plano-convex lens is applied to replace the PB phase lens to get the perfect vector beams. Experimental results are displayed in Fig. 5. Three different polarization orders *q* = 1, 2, 3 are selected to examine the validity of our scheme. Polarizer analysis shows that the correct polarizations as theoretical predications are generated.

In conclusion, we designed a series of Pancharatnam-Berry phase elements to produce the perfect vortex and vector beams. The structures of Pancharatnam-Berry phase elements are derived and improved from the conventional refractive perfect vortex generating system. Moreover, by combining the advantages of PB phase elements and refractive elements, we developed the scheme to generate perfect vector beams. Well agreements are presented in our experiments. The observed intensities are independent on the topological charges and polarization orders. Both of perfect vortex and vector beams are the candidate sources for large-capacity communication, high resolution imaging, and many other potential applications. Our scheme may provide the versatile solutions for integrated optics and fiber optics.

## Methods

### Sample preparation

Our PB phase elements are fabricated using femtosecond laser writing in fused silicon glass boards. With certain level intense femtosecond laser irradiation, sub-wavelength period grating oriented perpendicular to the polarization of laser will be formed spontaneously in glass^40^. Thus, the artificial birefringence is introduced to mould the polarization of light. As known, the principle optical axes are parallel or orthogonal to the subwavelength grating, thus the orientation of local optical axes can be artificially engineered by controlling the polarization of writing laser. Additionally, other than structures wrote pixel by pixel in general cases, these self-assembled structures can be written continuously, which means higher transform efficiency can be accessed. To get the PB phase samples, we firstly found out all the elements required to generate PV beams. Then, as all the elements are phase manipulating devices, their phase distributions are mathematically described. After that, according to the relationship between the orientation of local optical axes and the induced PB phase, the distribution of local optical axes can also be well described. Finally, all the elements are fabricated according to the derivation. Our samples are constructed for the operating wavelength 632.8 *nm*, efficient diameter ≥6 *mm* (Altechna R&D).

### Experimental measurements

The schematic of experiment setup is shown in Fig. 2. All the optical elements are cascaded to produce the PV beams. In experiments, a He-Ne laser operating in wavelength 632.8 *nm* (17 mW, Thorlabs HNL210L-EC) served as the optical source. The combination of linear polarizer and quarter-waveplate was used to prepare the polarization state of incidence. A CCD (charge-coupled device, Coherent LaserCam HR) camera was applied to record all the intensities.

## Additional Information

**How to cite this article:** Liu, Y. *et al*. Generation of perfect vortex and vector beams based on Pancharatnam-Berry phase elements. *Sci. Rep.* **7**, 44096; doi: 10.1038/srep44096 (2017).

**Publisher's note:** Springer Nature remains neutral with regard to jurisdictional claims in published maps and institutional affiliations.

## References

[b1] Allen L., Beijersbergen M. W., Spreeuw R. J. & Woerdman J. P. Orbital angular momentum of light and the transformation of Laguerre-Gaussian laser modes. Phys. Rev. A 45, 8185 (1992). 9906912 10.1103/physreva.45.8185

[b2] Grier D. G. A revolution in optical manipulation. Nature 424, 810 (2003). 12917694 10.1038/nature01935

[b3] Ostrovsky A. S., Rickenstorff-Parrao C. & Arrizón V. Generation of the “perfect” optical vortex using a liquid-crystal spatial light modulator. Opt. Lett. 38, 534 (2013). 23455127 10.1364/OL.38.000534

[b4] Vaity P. & Rusch L. Perfect vortex beam: Fourier transformation of a Bessel beam. Opt. Lett. 40, 597 (2015). 25680159 10.1364/OL.40.000597

[b5] García-García J., Rickenstorff-Parrao C., Ramos-García R., Arrizón V. & Ostrovsky A. S. Simple technique for generating the perfect optical vortex. Opt. Lett. 39 5305 (2014). 26466257 10.1364/OL.39.005305

[b6] Arrizón V., Ruiz U., Sánchez-de-la-Llave D., Mellado-Villaseñor G. & Ostrovsky A. S. Optimum generation of annular vortices using phase diffractive optical elements. Opt. Lett. 40, 1173 (2015). 25831285 10.1364/OL.40.001173

[b7] Banerji A., Singh R. P., Banerjee D. & Bandyopadhyay A. Generating a perfect quantum optical vortex. Phys. Rev. A 94, 053838 (2016).

[b8] Chen M., Mazilu M., Arita Y., Wright E. M. & Dholakia K. Dynamics of microparticles trapped in a perfect vortex beam. Opt. Lett. 38, 4919 (2013). 24322166 10.1364/OL.38.004919

[b9] Jabir M. V., Chaitanya N. A., Aadhi A. & Samanta G. K. Generation of “perfect” vortex of variable size and its effect in angular spectrum of the down-converted photons. Sci. Rep. 6, 21877 (2016). 26912184 10.1038/srep21877 PMC4766512

[b10] Li P. . Generation of perfect vectorial vortex beams. Opt. Lett. 41, 2205 (2016). 27176963 10.1364/OL.41.002205

[b11] Zhang C., Min C. & Yuan X. C. Shaping perfect optical vortex with amplitude modulated using a digital micro-mirror device. Opt. Commun. 381, 292 (2016).

[b12] Paez-Lopez R., Ruiz U., Arrizón V. & Ramos-Garcia R. Optical manipulation using optimal annular vortices. Opt. Lett. 41, 4138 (2016). 27607992 10.1364/OL.41.004138

[b13] Reddy S. G. . Non-diffracting speckles of a perfect vortex beam. J. Opt. 18, 055602 (2016).

[b14] Pancharatnam S. Generalized theory of interference and its applications. Proc. Indian Acad. Sci. A 44, 247 (1956).

[b15] Berry M. V. Quantal phase factors accompanying adiabatic changes. Proc. R. Soc. Lond. A 392, 45 (1984).

[b16] Lin D., Fan P., Hasman E. & Brongersma M. L. Dielectric gradient metasurface optical elements. Science 345, 298 (2014). 25035488 10.1126/science.1253213

[b17] Liu Y., Ke Y., Luo H. & Wen S. Photonic spin Hall effect in metasurfaces: a brief review. Nanophotonics 6, 51 (2017).

[b18] Hasman E., Kleiner V., Biener G. & Niv A. Polarization dependent focusing lens by use of quantized Pancharatnam-Berry phase diffractive optics. Appl. Phys. Lett. 82, 328 (2003).

[b19] Bomzon Z., Biener G., Kleiner V. & Hasman E. Space-variant Pancharatnam CBerry phase optical elements with computer-generated subwavelength gratings. Opt. Lett. 27, 1141 (2002). 18026387 10.1364/ol.27.001141

[b20] Biener G., Niv A., Kleiner V. & Hasman E. Formation of helical beams by use of Pancharatnam-Berry phase optical elements. Opt. Lett. 27, 1875 (2002). 18033387 10.1364/ol.27.001875

[b21] Marrucci L., Manzo C. & Paparo D. Pancharatnam-Berry phase optical elements for wave front shaping in the visible domain: Switchable helical mode generation. Appl. Phys. Lett. 88, 221102 (2006).

[b22] Huang L. . Three-dimensional optical holography using a plasmonic metasurface. Nat. Commun. 4, 2808 (2013).

[b23] He Y. . Higher-order laser mode converters with dielectric metasurfaces. Opt. Lett. 40, 5506 (2015). 26625037 10.1364/OL.40.005506

[b24] Mehmood M. Q. . Visible-frequency metasurface for structuring and spatially multiplexing optical vortices. Adv. Mater. 28, 2533 (2016). 26833667 10.1002/adma.201504532

[b25] Arlt J. & Dholakia K., Generation of high-order Bessel beams by use of an axicon. Opt. Commun. 177, 297 (2000).

[b26] Beijersbergen M. W., Coerwinkel R. P. C., Kristensen M. & Woerdman J. P. Helical-wavefront laser beams produced with a spiral phaseplate. Opt. Commun. 112, 321 (1994).

[b27] Zhan Q. Cylindrical vector beams: from mathematical concepts to applications. Adv. Opt. Photon 1, 1 (2009).

[b28] Zhan Q. Trapping metallic Rayleigh particles with radial polarization. Opt. Express 12, 3377 (2004). 19483862 10.1364/opex.12.003377

[b29] Zhan Q. Evanescent Bessel beam generation via surface plasmon resonance excitation by a radially polarized beam. Opt. Lett. 31, 1726 (2006). 16688275 10.1364/ol.31.001726

[b30] Abouraddy A. F. & Toussaint K. C. Three-dimensional polarization control in microscopy. Phys. Rev. Lett. 96, 153901 (2006). 16712157 10.1103/PhysRevLett.96.153901

[b31] Novotny L., Beversluis M. R., Youngworth K. S. & Brown T. G. Longitudinal Field Modes Probed by Single Molecules. Phys. Rev. Lett. 86, 5251 (2001). 11384470 10.1103/PhysRevLett.86.5251

[b32] Mino T., Saito Y. & Verma P. Control of near-field polarizations for nanoscale molecular orientational imaging. Appl. Phys. Lett. 109, 041105 (2016).

[b33] Li X. Cao Y. & Gu M. Superresolution-focal-volume induced 3.0 Tbytes/disk capacity by focusing a radially polarized beam. Opt. Lett. 36, 2510 (2011). 21725461 10.1364/OL.36.002510

[b34] Drevinskas R. . Laser material processing with tightly focused cylindrical vector beams. Appl. Phys. Lett. 108, 221107 (2016).

[b35] Pfeiffer C. & Grbic A. Controlling Vector Bessel Beams with Metasurfaces. Phys. Rev. Applied 2, 044012 (2014).

[b36] Chen Y., Wang F., Yu J., Liu L. & Cai Y. Vector Hermite-Gaussian correlated Schell-model beam. Opt. Express 24, 15232 (2016). 27410801 10.1364/OE.24.015232

[b37] Fu S. Zhang S. & Gao C. Bessel beams with spatial oscillating polarization. Sci. Rep. 6, 30765 (2016). 27488174 10.1038/srep30765 PMC4973278

[b38] Milione G., Sztul H. I., Nolan D. A. & Alfano R. R. Higher-order Poincaré sphere, stokes parameters, and the angular momentum of light. Phys. Rev. Lett. 107, 053601 (2011). 21867067 10.1103/PhysRevLett.107.053601

[b39] Liu Y. . Realization of polarization evolution on higher-order Poincaré sphere with metasurface. Appl. Phys. Lett. 104, 191110 (2014).

[b40] Beresna M., Gecevičius M. & Kazansky P. G. Polarization sensitive elements fabricated by femtosecond laser nanostructuring of glass. Opt. Mater. Express 1, 783 (2011).

